# Learning From Older Adults to Promote Independent Physical Activity Using Mobile Health (mHealth)

**DOI:** 10.3389/fpubh.2021.703910

**Published:** 2021-08-12

**Authors:** Camille Nebeker, Zvinka Z. Zlatar

**Affiliations:** ^1^Design Lab and Research Center for Optimal Digital Ethics in Health (ReCODE Health), Herbert Wertheim School of Public Health and Human Longevity Science, University of California, San Diego, La Jolla, CA, United States; ^2^Wellness Initiative for Senior Enrichment (WISE) Lab, Department of Psychiatry, University of California, San Diego, La Jolla, CA, United States

**Keywords:** mobile health, digital health, physical activity, interventions, older adults, research design, participant perspectives, research ethics

## Abstract

**Background:** Healthy aging is critically important for several reasons, including economic impact and quality of life. As the population of older adults rapidly increases, identifying acceptable ways to promote healthy aging is a priority. Technologies that can facilitate health promotion and risk reduction behaviors may be a solution, but only if these mobile health (mHealth) tools can be used by the older adult population. Within the context of a physical activity intervention, this study gathered participant's opinions about the use of an mHealth device to learn about acceptance and to identify areas for improvement.

**Methods:** The Independent Walking for Brain Health study (NCT03058146) was designed to evaluate the effectiveness of a wearable mHealth technology in facilitating adherence to a physical activity prescription among participants in free-living environments. An Exit Survey was conducted following intervention completion to gauge participant's perceptions and solicit feedback regarding the overall study design, including exercise promotion strategies and concerns specific to the technology (e.g., privacy), that could inform more acceptable mHealth interventions in the future. The Digital Health Checklist and Framework was used to guide the analysis focusing on the domains of Privacy, Access and Usability, and Data Management.

**Results:** Participants (*n* = 41) were in their early 70's (mean = 71.6) and were predominantly female (75.6%) and White (92.7%). Most were college educated (16.9 years) and enjoyed using technology in their everyday life (85.4%). Key challenges included privacy concerns, device accuracy, usability, and data access. Specifically, participants want to know what is being learned about them and want control over how their identifiable data may be used. Overall, participants were able to use the device despite the design challenges.

**Conclusions:** Understanding participant's perceptions of the challenges and concerns introduced by mHealth is important, as acceptance will influence adoption and adherence to the study protocol. While this study learned from participants at studycompletion, we recommend that researchers consider what might influence participant acceptance of the technology (access, data management, privacy, risks) and build these into the mHealth study design process. We provide recommendations for future mHealth studies with older adults.

## Introduction

The population of older adults, defined as those age 60 and over, is rapidly increasing. Today, older adults comprise about 12% of the global population yet, this figure is expected to double over the next few decades reaching 22% by 2050 (1). Keeping our aging population healthy and able to live independently is a major public health priority (2). In fact, the World Health Organization defined healthy aging as “the process of developing and maintaining the functional ability that enables well-being in older age” (3). Factors that support healthy aging are grounded to the individual's capacity and environmental characteristics. Individual capacity is influenced by their mental and physical abilities, which are affected by overall health status [e.g., disease, injuries; (3)]. The impact of physical activity on health is well-established with numerous studies documenting its beneficial effects on cardiovascular disease, depression, cognitive and brain health (4–10).

The development of technologies to support healthy aging efforts are on the rise. These technologies include wearable and remote pervasive sensors, voice activated systems, and predictive analytics, including digital phenotyping (11–16). Digital tools and strategies are increasingly applied to health promotion, disease prevention, and treatment efforts yet, are not always tested with diverse populations. For example, researchers found that mobile health or “mHealth” studies that involved vulnerable populations revealed concerns about participant privacy and data sharing practices (17). When working with older adults, as well as others who are not digital natives, the need to consider barriers and facilitators to access and usability of the technology is important. By knowing what the potential barriers are, the technologies can be designed for access and are then more likely to be adopted and used (18).

**Figure 1:**
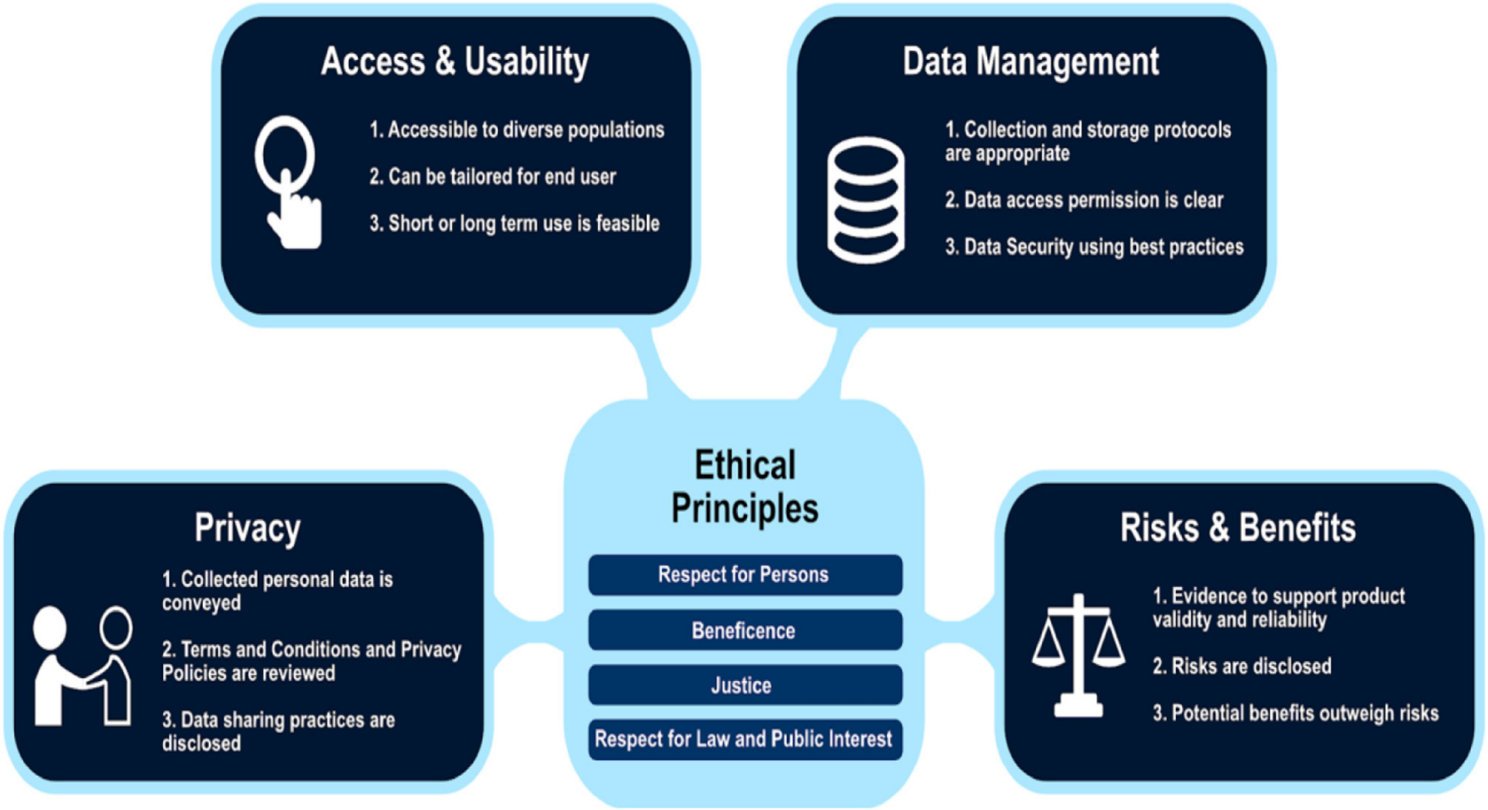
Digital Health Checklist and the four domains connected to foundational ethical principles used with permission. Published with permission of C. Nebeker, ReCODE Health The DHC-R is licensed under a Creative Commons Attribution-Non-Commercial 4.0 International License (2018–2020) and available at https://recode.health/tools/.

This study employed an Exit Survey to gather older adult's opinions about the use of an mHealth device after participating in a randomized controlled trial to promote physical activity in free living environments. We employed the Digital Health Checklist (DHC) and Framework (19) to guide our analysis of participant's perceptions about the use of mHealth. The DHC framework was developed in 2019 in response to uncertainties that the mHealth research community (i.e., technologists, researchers, ethicists, regulators, institutions, participants involved in the digital health research process, and other stakeholders) voiced specific to navigating new privacy, data management, and risk assessment challenges (11). The DHC is grounded in four accepted ethical principles in biomedical and behavioral research: respect for persons, beneficence, justice, and respect for law and public interest (19). The ethical principles connect with four orthogonal domains that are of critical importance in digital health research: (1) *Access and Usability*, (2) *Risks and Benefits*, (3) *Privacy*, and (4) *Data Management* (Figure 1). This study focused on the following DHC domains: *Access and Usability, Privacy, and Data Management*. Based on participant's responses to the Exit Survey, we also provide recommendations for researchers conducting mHealth trials with older adults. Lessons learned from participants will help guide future mHealth interventions to improve device adoption and adherence.

## Materials and Methods

### Study Design

The study was a small 12-week randomized controlled trial (NCT03058146: Independent Walking for Brain Health) designed to test the feasibility of using an mHealth device to assist older adults independently achieve and maintain physical activity guidelines (≥150 min per week of moderate intensity physical activity) in their free-living environments to improve brain health. Participants were randomly assigned to a Physical Activity (*n* = 21) or a Healthy Aging Education condition (*n* = 23). Random assignment was stratified by age and sex. Only the participants randomized to the Physical Activity condition received an individualized exercise prescription and a commercially available mHealth device. The mHealth device was a heart rate tracker that could be programmed through its app to set custom heart rate target zones equivalent to moderate physical activity intensity for each person. The device was programed to vibrate and flash different colored lights (blue = below zone, green = in zone, red = above zone) when participants were deviating from their individually prescribed heart rate target zones. Participants did not need to have their smartphone with them during the exercise sessions, since data gathered from the mHealth device was later synced with the app and emailed to the study team. This helped with accountability and tracking by the study team to provide weekly feedback about progress via email and phone calls. Upon completion of the study, an online Exit Survey was deployed to gather participant's opinions about study participation and thoughts about the use of mHealth devices to inform future mHealth interventions.

### Participants

Participants who completed the Exit Survey were 41 out of the 44 randomized to the intervention (Physical Activity condition: *n* = 20/21, Healthy Aging Education condition: *n* = 21/23). They were all community-dwelling, English-speaking older adults between the ages of 65 and 80 years. They were recruited from the University of California San Diego's Shiley-Marcos Alzheimer's Disease Research Center longitudinal study of aging, from ResearchMatch (https://www.researchmatch.org), flyers, community engagement talks (i.e., retirement communities, senior centers, libraries, health fairs), and by word of mouth. Participants did not have cognitive impairment as defined by scores >1 standard deviation below age appropriate norms on the Mattis Dementia Rating Scale (MDRS) (20). Participants who met the following criteria were included in the study: ability to obtain a signed physician's clearance to engage in physical activity, be insufficiently active (<150 min of moderate intensity physical activity per week), no contraindications for magnetic resonance imaging (MRI), ambulatory and able to walk independently. Exclusion criteria included: mild cognitive impairment or dementia based on standard neuropsychological testing [i.e., those with two or more scores <1 standard deviation from age-appropriate norms within one or more cognitive domains were excluded; (21)], history of head injury with loss of consciousness within the past 6 months and/or history of severe traumatic brain injury, major neurologic or psychiatric disorders (i.e., bipolar disorder, schizophrenia, Parkinson's disease), history of major vascular events (i.e., myocardial infarction, stroke), diabetes, poorly controlled medical conditions, and history of falls in the past year resulting in hospitalization.

### Randomized Controlled Trial Description

The Independent Walking for Brain Health study was a 12-week, unsupervised mHealth intervention to promote brain health in older adults. All potential participants were pre-screened via telephone to determine basic eligibility and to obtain physician's clearance. For those meeting basic eligibility criteria, a baseline appointment was scheduled and an Actigraph accelerometer (wGT3X-BT) was mailed to them to measure baseline physical activity for 7-days. Participants brought the accelerometer with them to their baseline appointment, at which time cognitive testing and a sub-maximal treadmill assessment were conducted. If participants met the cognitive criteria and completed the treadmill test successfully, they were scheduled for their randomization visit. During the randomization visit, participants underwent a baseline brain MRI, were informed of their group assignment, and were provided with either a walking or a reading prescription. All aspects of the intervention and a tutorial on how to use the mHealth device (for those assigned to the Physical Activity condition) were explained during this visit. Participants were then sent home with printed materials including their prescriptions, upcoming appointment schedule, and upcoming monitoring phone call schedule. All aspects of the intervention took place remotely aside from the measurement visits (baseline, mid, and post-intervention). There were weekly monitoring phone calls during the first month, followed by bi-weekly phone calls during months 2 and 3. The intervention was individualized, and enrollment was ongoing from 2017 to 2020.

Individualized target heart rate zones to achieve moderate intensity activity were derived from sub-maximal fitness testing on a treadmill during the baseline session and corresponded to a heart rate equivalent to at least 3.3 metabolic equivalents (METs). Participants were instructed to maintain moderate intensity activity by following the mHealth device prompts and attempting to walk above their minimum prescribed heart rate during each exercise session. This “just in time” feedback was hypothesized to help older adults achieve and maintain moderate levels of physical activity independently when walking in their free-living environments.

### Exit Survey Description and Implementation Process

This article focuses on the Exit Survey used to understand participant's motivations and perceptions. Specifically, we wanted to know why they would enroll in studies promoting brain health and their opinions regarding the use of mHealth tools within this context. The purpose of the Exit Survey was to use the information gathered to improve the design of future mHealth trials.

The Exit Survey consisted of several multiple choice, forced choice, and open-ended questions about the following topics: motivation, participation experience, beliefs about brain health, effectiveness of different intervention techniques, mHealth device use, challenges, and utility in helping to achieve physical activity goals, and data privacy and confidentiality. Items were developed by the study investigators based on areas considered to be important in the design of mHealth interventions generally and, more specifically when conducting research to promote brain health with older adults. By learning about participant experiences, we can improve the study protocol to account for issues that may be problematic or impede adherence. The Exit Survey was sent via REDCap (https://www.project-redcap.org/) to participants the day after completion of the post-intervention visit. Some participants who were randomized but did not finish the intervention (*n* = 3, 7.3%) completed the Exit Survey and their responses were included in this analysis.

### Analysis

We present descriptive data based on the Exit Survey to characterize participant's responses and provide useful recommendations for future interventions using just in time adaptive feedback to improve brain health in aging. For continuous variables, we present means and standard deviations. For forced choice and multiple-choice questions, we present percentages. Exit Survey items were organized based on alignment with the framework undergirding the Digital Health Checklist (DHC), which was developed to help researchers design digital health studies (19).

The Exit Survey created for this study was developed before the DHC was published and, as such, was only able to be applied retrospectively. That said, the survey included three of the four domains explicitly (*Access and Usability, Privacy, and Data Management*). The domain of *Risks and Benefits*, while not explicit in the Exit Survey, was acknowledged in questions that pertained to motivation to exercise and the benefits of being more consistently active as well as risks associated with unauthorized access to personal health data. In this manuscript, we focus on *Access and Usability, Privacy, and Data Management* (refer to Figure 1). To organize the Exit Survey results, items were re-arranged *post-hoc* according to alignment with the four domains.

#### Access and Usability

The Access and Usability domain prompts consideration about the extent to which a digital health product is designed and selected. The overarching goal is to ensure that participants who will be asked to use the product are able to use it as intended.

#### Privacy

The domain of Privacy reflects the type of personal information and/or health-related data collected and expectations of the participant to keep information secure or, if shared, accessible information about how and with whom data are shared. Privacy also considers the rights of bystanders who may be in proximity of a research participant and who may be unintentionally included in the data set.

#### Data Management

Data Management is a domain that spans collection, storage, and sharing of information obtained during the study. Extending and somewhat overlapping with privacy, Data Management takes into account who owns data, how data are shared including to what extent data are incorporated into other systems (interoperability) and secured (encryption standards, and compliance with existing regulations).

## Results

See Table 1 for participant characteristics. Participants were in their early 70's (mean = 71.6) and were predominately female (75.6%) and White (92.7%). Most were college educated (16.9 years) and all of them had normal cognitive functioning. More than half of participants in this study (65.9%, *n* = 27) reported having used a fitness device in the past, with 85.4% (*n* = 35) stating that they enjoyed using technologies (e.g., online calendar, reminders, smartphone apps) in their everyday life.

**Table 1:** Participant characteristics.

	**Physical activity (*n* = 20)**	**Healthy aging education (*n* = 21)**	**Total (*n* = 41)**
Age (years)	71.2 (4.38)	71.95 (3.95)	71.59 (4.13)
Education (years)	16.65 (2.43)	17.05 (2.1)	16.85 (2.25)
% female	75	76.2	75.6
% Hispanic/Latino	10	4.8	7.3
MDRS total raw score	140.35 (2.35)	138.95 (3.47)	139.63 (3.02)
MDRS total T-score	54.3 (3.26)	52.43 (5.08)	53.34 (4.35)

*Demographic characteristics of randomized participants who completed the Exit Survey. Values represent the mean (standard deviation) unless otherwise stated. MDRS, Mattis Dementia Rating Scale*.

### Access and Usability

**Figure 2:**
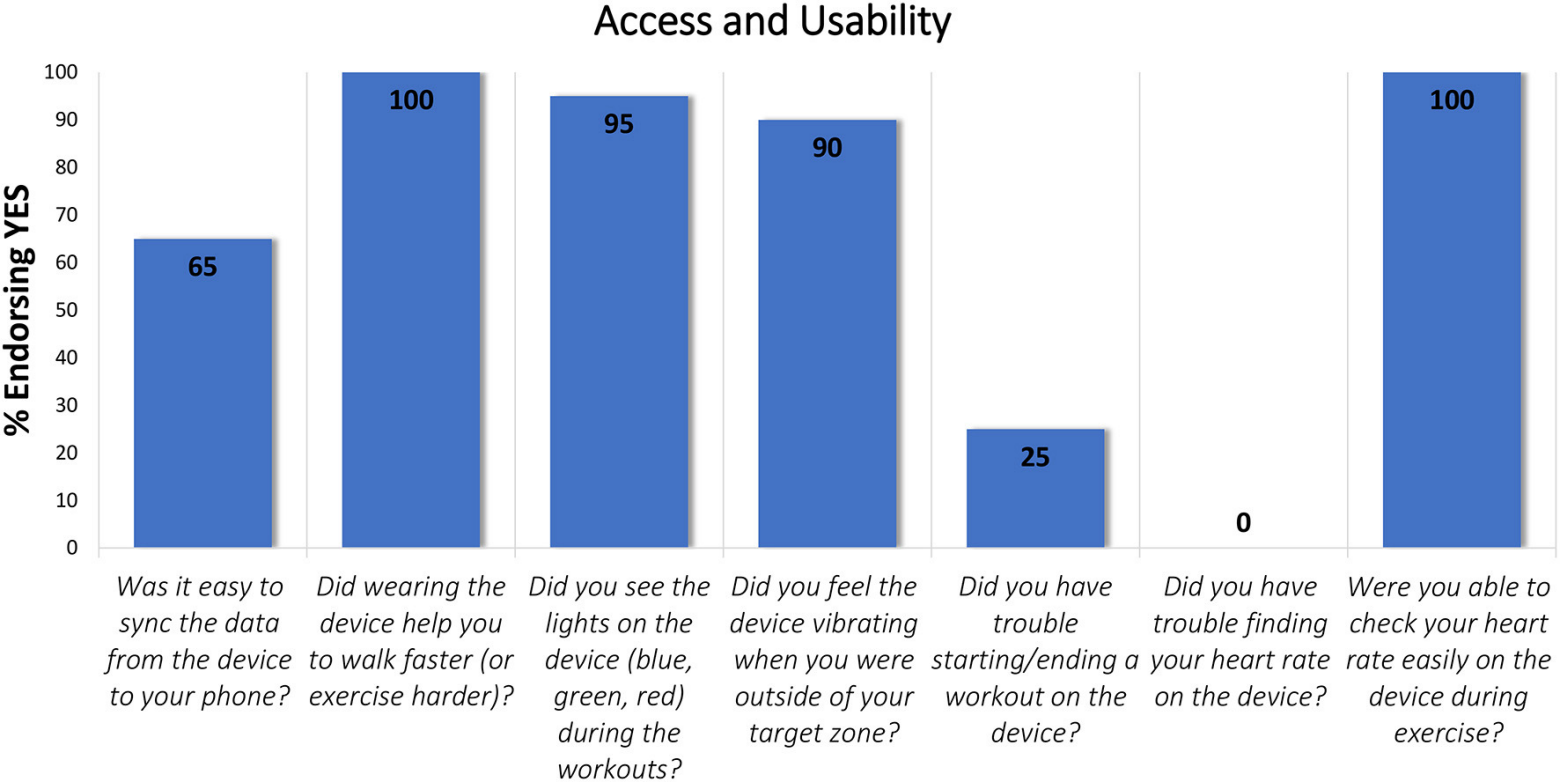
Exit Survey items that aligned with the Access and Usability domain.

Seven items within the Exit Survey aligned with the Access and Usability domain (Figure 2) and, unlike the other survey items, were only answered by those assigned to the Physical Activity condition (*n* = 20).

All participants reported that the device helped them to walk faster with 65% stating it was easy to sync the data from the device to their phone. The light and vibration signals on the device are designed to draw the user's attention to their goals and, potentially motivate the person wearing the device to adapt their walking pace accordingly. Ninety percent of participants indicated that they could feel the vibration signal during their workouts and 95% reported seeing the light signal during exercise. When starting or stopping a workout on the device, 25% reported having trouble. With respect to “Checking heart rate easily on the device during exercise” 100% of participants in the Physical Activity condition (*n* = 20) indicated that they could easily check their heart rate on the device.

An open-ended question asked participants if they would wear the device in the future and 60% (*n* = 12) of them said yes. If they answered “no” to this question (40%, *n* = 8), they were asked why they would not wear the study device in the future. Responses focused on physical attributes, concerns about accuracy, technical issues, and difficulty operating the device. For example, some said the device was cumbersome to wear and not intuitive to use, that they disliked the wrist band or found it too bulky. Others questioned the accuracy and indicated they had technical difficulties using the device (i.e., with the syncing process or how to start and record a workout). Other participants stated that they bought a different device after the study ended or that they learned how to maintain their heart rate target without a device.

### Privacy

**Figure 3:**
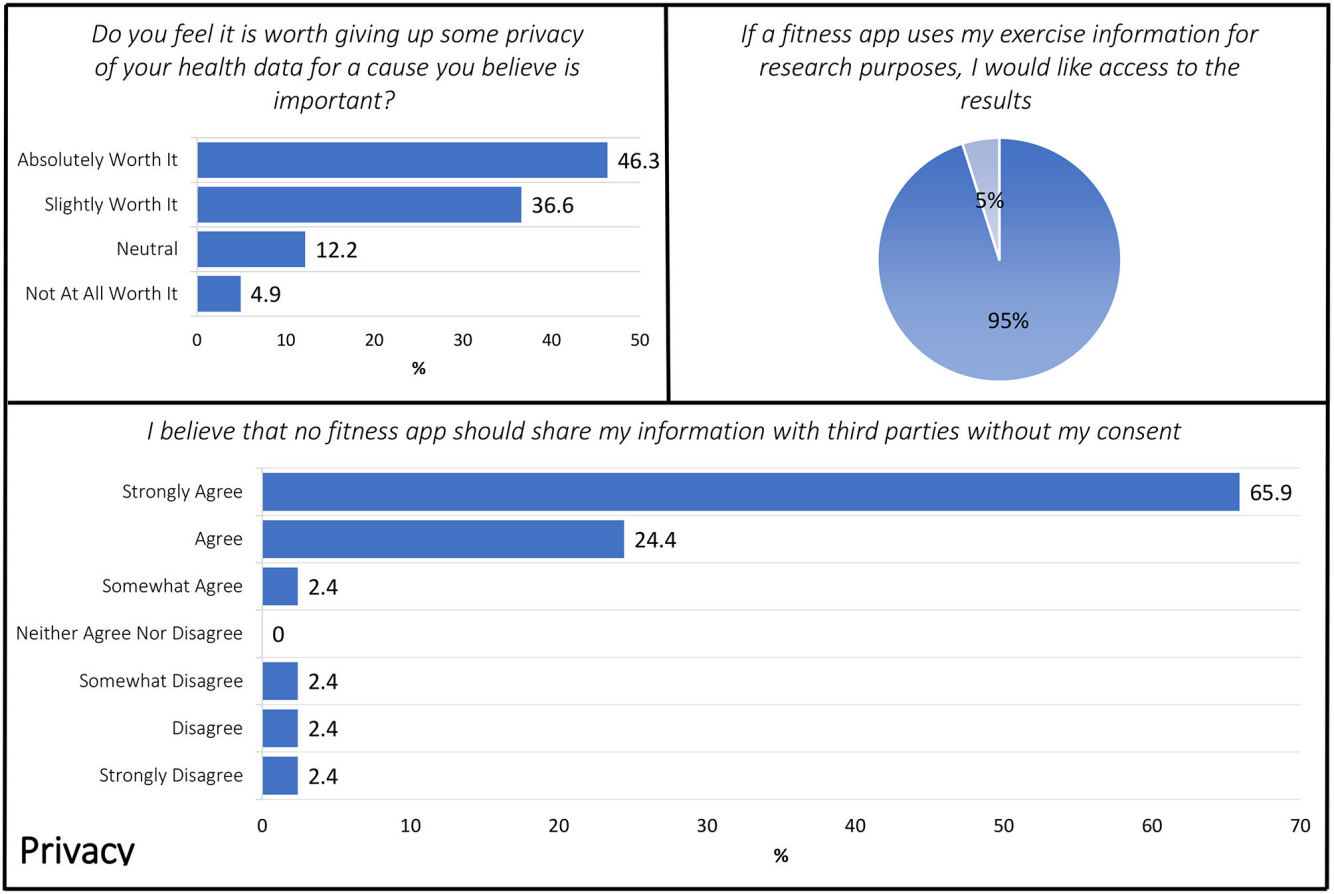
Exit Survey items that aligned with the Privacy domain.

Three items included in the Exit Survey aligned with the DHC Privacy domain including: (1) Do you feel it is worth giving up some privacy of your health data for a cause you believe is important (in this case, to advance cognitive aging science)? (2) If a fitness app uses my exercise information for research purposes, I would like access to the results. (3) I believe that no fitness app should share my information with third parties without my consent (Figure 3).

The ethical principle of “respect for persons” is generally demonstrated in biomedical and behavioral research via an informed consent process. Respect is also demonstrated by how a researcher or research organization shares information about the individual and results stemming from a study. Within this context, the Exit Survey included several items about when permissions or consent would be needed or desired with respect to sharing of personal information. When asked if they thought it “is worth giving up some privacy” of personal health data for causes they believed to be important, 46.3 (*n* = 19) responded that it is “absolutely worth it” with only 4.9% (*n* = 2) stating it was “not at all worth it” and 36.6 (*n* = 15) with a “slightly worth it” perspective. When asked about preferences to have access to study results if their fitness data were used for research purposes, respondents were clearly interested in having access to study results with 95% (*n* = 39) stating that they wanted access. Similarly, 92.7% of the sample agreed to some extent that no fitness app should share their information with third parties without their consent.

### Data Management

**Figure 4:**
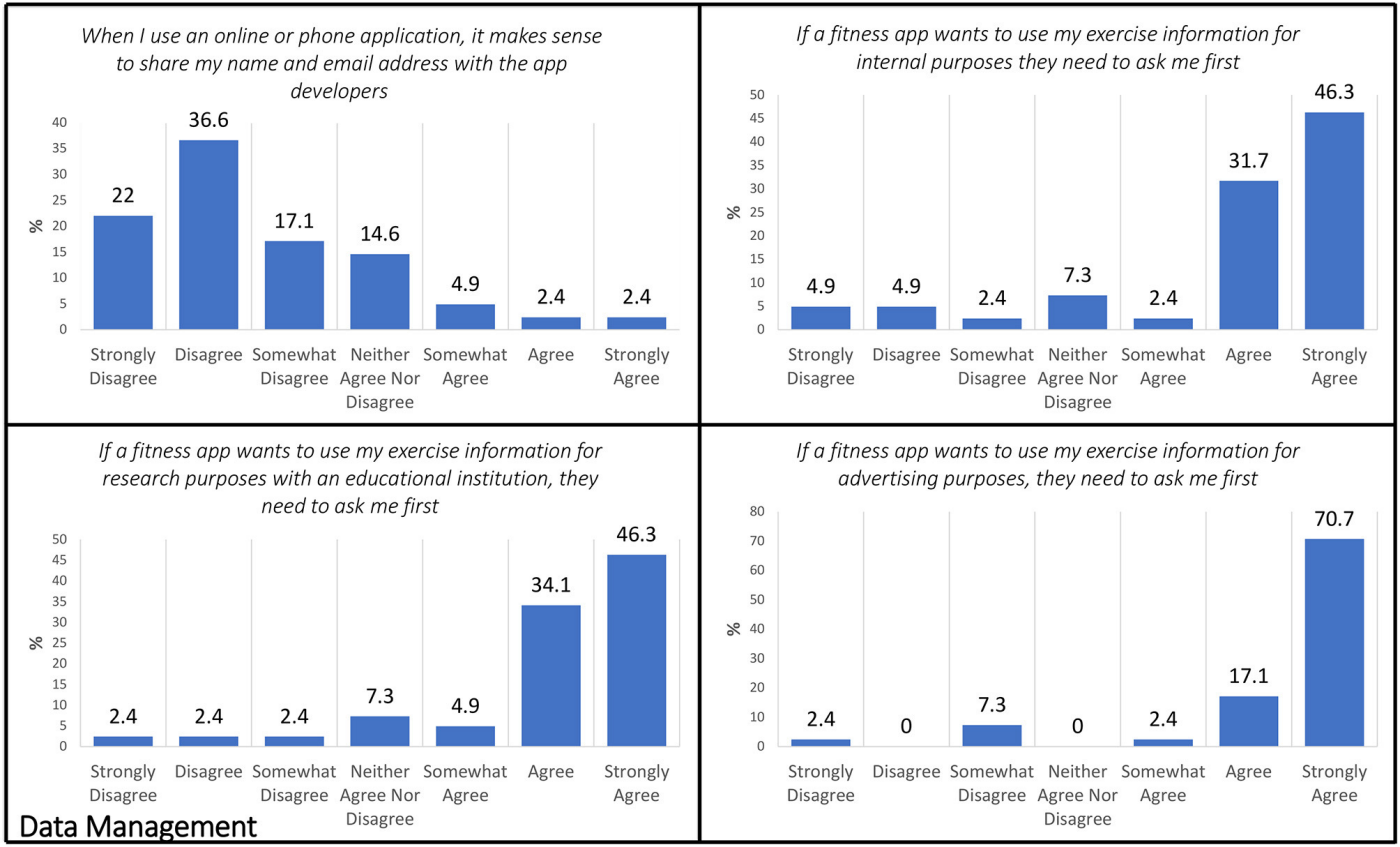
Exit Survey items that aligned with the Data Management domain.

Within the Exit Survey, the following five items aligned with the Data Management domain: (1) When I use an online or phone application, it makes sense to share my name and email address with the app developers. (2) App developers should “de-identify” my app data (for example, if Fitbit uses my exercise information for internal purposes, they should use my data without my name or personal information attached to it). (3) If a fitness app wants to use my exercise information for internal purposes (i.e., app improvement, internal search), they need to ask me first. (4) If a fitness app wants to use my exercise information for advertising purposes, they need to ask me first. (5) If a fitness app wants to use my exercise information for research purposes with an educational institution, they need to ask me first (Figure 4).

When asked if it makes sense to share contact information (name and email) with the app developers, only 2.4% agreed or strongly agreed (*n* = 2) with 4.9% (*n* = 2) indicating some agreement, whereas a majority disagreed 36.6% (*n* = 15) with 17.1% (*n* = 7) and 22% (*n* = 9) indicating somewhat and strong disagreement, respectively. Subsequently, a minority were ambivalent with 14.6% (*n* = 6) neither agreeing nor disagreeing with the idea of app developers sharing contact information. However, if the app developers wanted to use potentially identifiable exercise information for internal purposes (e.g., to improve their product), 80.4% (*n* = 33) indicated that they wanted to be asked first with 46.3% of those choosing to “strongly agree” with that position. When then distinction was made to use personal level fitness data to advance health research by sharing with an academic institution, nearly half, 46.3% (*n* = 19) strongly agreed that they wanted to provide permission with only 7.2% (*n* = 3) in disagreement with an equal number (*n* = 3) responding with “neither agree nor disagree.” If the app developer wanted to use personal level data for advertising purposes, respondents expressed a definite desire to provide permission in advance with 70.7% strongly agreeing to this position and only 9.7% (*n* = 4) disagreeing.

**Figure 5:**
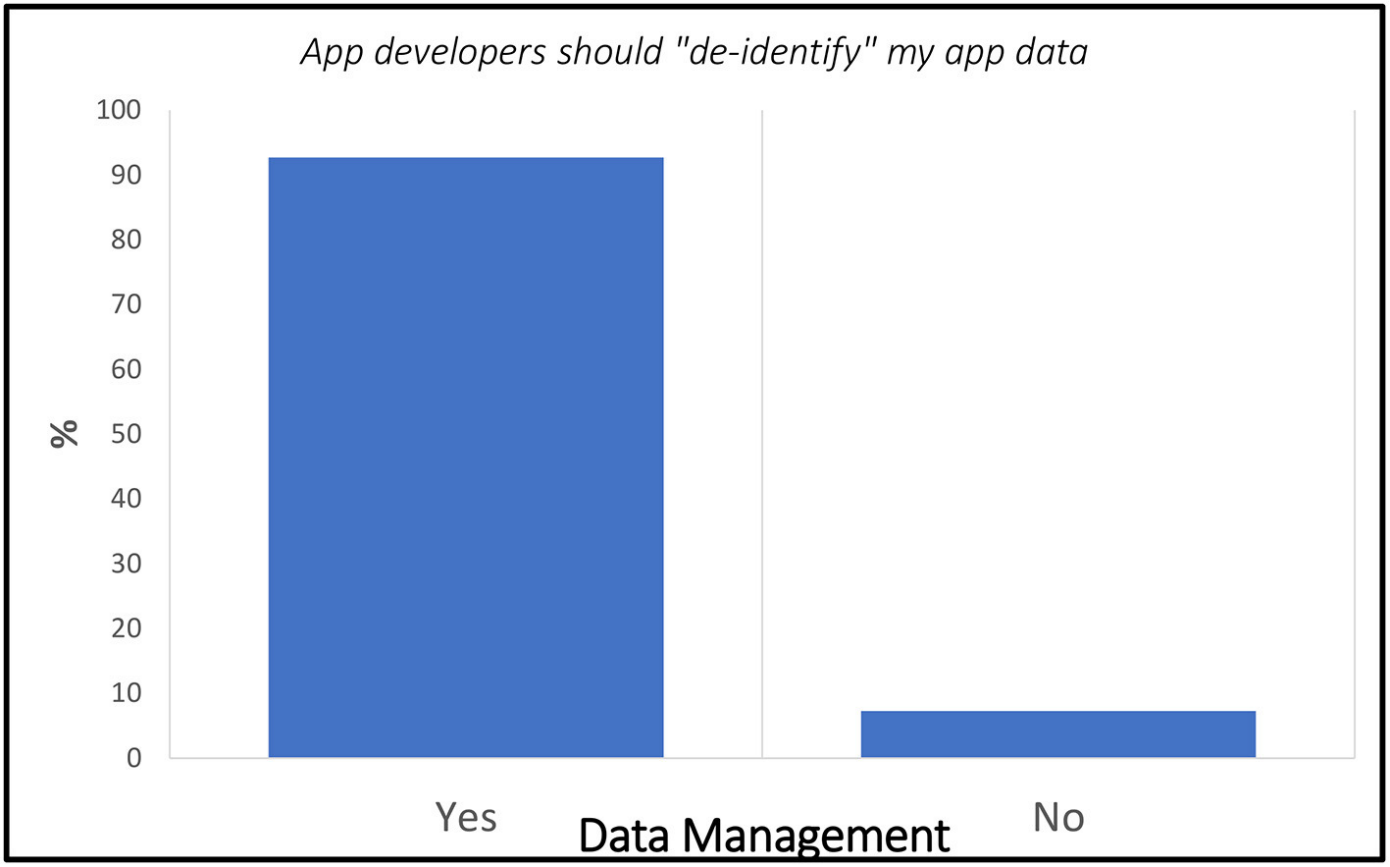
Exit Survey items that aligned with the Data Management domain.

Specific to the idea of “de-identified” personal fitness data being used by the app developer for internal purposes, a clear majority of 92.7% (*n* = 38) responded “yes” that information used for internal purposes should not be attached to personal information (Figure 5).

## Discussion

With the rapid growth of the older adult population, mobile, and wearable technologies have great potential to mitigate challenges and help maintain an independent lifestyle. Importantly, many in this demographic are “Baby Boomers” who are semi-literate when it comes to technology use and comfort because they have used technologies in both their professional and personal lives (22). Yet, if the technologies created are not inclusive of older adults during the conception and prototype development phase, it is likely the products may not be responsive to their needs. Currently, when these tools are selected for use in an mHealth research study, the researcher has minimal resources to identify which may be most appropriate for the task at hand, and this is especially true for researchers working with older adults. Although a basic “mHealth for older users” framework exists, it is just a first step toward advancing the need of older adults to adopt technology that is designed with them in mind (23–25).

To help fill this gap, Lewis and Neider (14) looked at the design features of wearable technologies and recommended that designers consider changes in cognition, motor, and sensory function when designing for older adults. This is similar to the MOLD-US framework, which identified the need for design guidelines to address cognitive, physical, and motivational barriers with older adults (24). Our results are in line with this guidance showing that some older adults thought our device was “cumbersome” and it was difficult to maneuver its wristband. The wristband had several holes that needed to be aligned with the buckle and then tightened for a secure fit. This requires fine motor skills and good sensory ability on the fingers to appropriately tighten the band. Participants also reported that they encountered “technical difficulties” and it was hard to start and end a workout session on the device, perhaps speaking to the cognitive involvement needed to successfully learn how to use the device. For this study, participants had to learn how to start and end a workout to record the heart rate during a physical activity session, then syncing the data with the app on their smartphone, and finally emailing the data file to the study team. This is a complex series of tasks that necessitates adequate cognition and familiarity with technology for successful performance. Although all our participants had cognition within normal limits and reported being comfortable using technology in everyday life (85.4%), some still experienced difficulties with this process. This further highlights the importance of designing with the older adult in mind to promote device adoption and adherence, a concern that has been expressed by older adults themselves (26).

Despite these challenges, all participants reported that the device helped them to walk faster and that they adjusted their walking pace accordingly as a result of the “just in time” feedback. On average, participants increased the time they spent in their individualized heart rate target zone by 95% (from 144.8 min/week to 282.4 min/week) over the course of 12 weeks. This reflects participant's motivation to try their best despite all the difficulties they experienced with the mHealth device and speaks volumes regarding their willingness to learn new technologies and face new challenges. A different study also found that older adults living with chronic pain were highly willing (85% of the sample) to use mHealth to manage their symptoms (26).

The most novel aspect of this study was our emphasis in obtaining participant's perceptions regarding privacy and data management within the context of the DHC framework. We learned that participants want to be asked permission when apps share their information and that they would like access to any results if a fitness app uses their information for research purposes. Moreover, they mostly disagreed with having to share their name and email address with app developers. For the most part, participants agreed that consent needs to be obtained from them by fitness apps if they want to use their data for internal, advertising, or research purposes. They also felt strongly that their fitness information needs to be de-identified. Interestingly, 83% of participants felt it is worth giving up some privacy of their health data for a cause they believe is important. This underscores that participants take into consideration the risk/benefit ratio of privacy and disclosure to make decisions about participating in research studies aligned with their aspirations.

For behavioral health scientists, the use of mobile apps and wearable sensor technologies to monitor and intervene with research participants is relatively novel, yet rapidly increasing (27). As with any emerging field, the terminology is evolving with terms like “mHealth,” “digital health,” and “eHealth” being used to describe biomedical and behavioral health research studies that utilize wearable or remote passive sensors to capture personal health data. In the early days of mobile health research, the sensors used were developed for research purposes whereby the researcher had access to, and control of the data collected from participants (e.g., wearable cameras like Sense Cam, actigraphy devices). However, with the release of wearable fitness products direct to consumer (e.g., Fitbit, Garmin, Apple), the scientific research community has increased its reliance on using these tools to advance mHealth research. Whether these tools produce reliable and valid data is important yet may be difficult to assess due to the proprietary nature of for-profit entities. Moreover, these devices are not designed with the older adult in mind. Health researchers may not have the resources or training to know what to look for when evaluating a specific technology or anticipate what features may pose problems for a research participant. The digital health framework and accompanying checklist were developed to fill this gap and provide researchers with a decision support aid to facilitate technology selection. Moreover, we present our recommendations below to help guide future mHealth studies with older adults based on the lessons learned from the participants themselves and from our study team. As such, the DHC (19) can be used in conjunction with existing mHealth design guidelines for older adults (23, 24, 27) and our recommendations to inform future device development and intervention design and bolster older adult's adherence to lifestyle mHealth interventions.

## Recommendations Based on Challenges Identified by the Participants

➢ Choose devices that are aesthetically pleasing.
➢ Consider a wristband that buckles easily, without the need for fine motor control.
➢ Use large buttons that are clearly visible rather than having to use touch to “feel” where a button is located on the device.
➢ Reduce participant burden by having the device automatically detect the behavior of interest so the participants do not have to manually enter it or initiate it.
➢ Choose a device that syncs automatically and directly to the app or database with ease.
➢ Choose devices that give the researcher and participant direct access to the data.

## Recommendations Based on Lessons Learned From the Study Team

➢ If using a commercially available device, get information about the design process and how they take into consideration the older adult's perspective.
➢ Test the device against a gold standard, if possible, to ensure it accurately measures what it purports to measure.
➢ Ensure the device you select will not be discontinued during the length of your study.
➢ Learn how the device company will share and/or use the information and communicate this to your participants during the informed consent process.
➢ Offer to create “fake” accounts for participants who do not feel comfortable providing their information to the app developers.

## Conclusion

This innovative study applied the DHC framework to guide analysis of older participants' opinions about the use of mHealth as part of a randomized controlled trial to increase physical activity and promote brain health. We learned about participants' perceptions regarding access/usability, privacy, and data management of mHealth use within the context of the DHC framework. Older adults want to be asked permission when fitness apps share their information, they want to know what is being learned about them and want control over how their identifiable data may be used. There were also concerns about the accuracy of the data gathered by the mHealth device and several challenges associated with device wear, which should be taken into consideration in future mHealth studies that involve older adults. Importantly, participants reported that the mHealth device helped them improve their walking speed, despite all the challenges they experienced. A key takeaway of this study is that older adults are willing and able to use mHealth in research settings, expressed a desire to be involved in how their data is collected and shared, and take into consideration the risk/benefit ratio of privacy and disclosure of information to make decisions about participating in research studies aligned with their aspirations. Research participants are an excellent resource and should be involved during the planning stages of mHealth studies to avoid potential mistakes. Learning about unique participant perspectives regarding privacy, data confidentiality, desire to provide permission, and sharing study results are all valid factors that should be considered when designing mHealth research.

## Strengths and Limitations

This study is unique in its capturing of participants' perceptions about privacy and data management concerns related to the use of mHealth. Moreover, the use of the DHC framework to guide our analysis is a step forward toward advancing research in mHealth interventions. One limitation was the collection of the Exit Survey at the end of the study rather than before device selection. We did this to learn about participants' perceptions of device usability and their thoughts about participation in the intervention to inform future research. Future studies should gather older adult's perspectives prior to choosing a device or ask them to collaborate in the process of choosing one to improve device adoption. Another limitation of this study is its small sample size and its homogeneous nature (mostly White, well-educated, English-speaking older women). As such, our findings cannot be generalized to more diverse older adult samples. Given the increasingly diverse and growing nature of the older adult population (28), it will be important for future research in this area to obtain opinions about mHealth use and privacy from larger and more diverse samples that are representative of the United States older adult population. Since mHealth research with diverse populations has lagged, engaging diverse older adults in mHealth clinical trials to prevent cognitive decline is of outmost importance (29) to ensure they are represented in research and their clinical care improved.

## Data Availability Statement

The raw data supporting the conclusions of this article will be made available by the authors, without undue reservation.

## Ethics Statement

The studies involving human participants were reviewed and approved by University of California San Diego Human Research Protections Program. The patients/participants provided their written informed consent to participate in this study.

## Author Contributions

ZZ designed the parent study, obtained funding, conducted the study, conceptualized the current study, analyzed the data, and co-wrote the manuscript. CN assisted with the development of the Exit Survey items related to privacy and data management, helped to guide the analysis and conceptualization through the DHC framework, and co-wrote the manuscript. ZZ and CN provided critical feedback and helped shape the research, analysis, and manuscript. All authors contributed to the article and approved the submitted version.

## Author Disclaimer

The content is solely the responsibility of the authors and does not necessarily represent the official views of the National Institutes of Health. Study data were collected and managed using REDCap electronic data capture tools hosted at the University of California, San Diego CTRI [UL1TR001442].

## Conflict of Interest

The authors declare that the research was conducted in the absence of any commercial or financial relationships that could be construed as a potential conflict of interest.

## Publisher's Note

All claims expressed in this article are solely those of the authors and do not necessarily represent those of their affiliated organizations, or those of the publisher, the editors and the reviewers. Any product that may be evaluated in this article, or claim that may be made by its manufacturer, is not guaranteed or endorsed by the publisher.
